# Transmembrane Prolines Mediate Signal Sensing and Decoding in Bacillus subtilis DesK Histidine Kinase

**DOI:** 10.1128/mBio.02564-19

**Published:** 2019-11-26

**Authors:** Pilar Fernández, Lucía Porrini, Daniela Albanesi, Luciano A. Abriata, Matteo Dal Peraro, Diego de Mendoza, María C. Mansilla

**Affiliations:** a Instituto de Biología Molecular y Celular de Rosario (IBR-CONICET), Rosario, Argentina; b Departamento de Microbiología, Facultad de Ciencias Bioquímicas y Farmacéuticas, Universidad Nacional de Rosario, Rosario, Argentina; c Institute of Bioengineering, École Polytechnique Fédérale de Lausanne, Lausanne, Switzerland; d Swiss Institute of Bioinformatics, Lausanne, Switzerland; Institut Pasteur

**Keywords:** histidine kinase, proline, thermosensing, two-component regulatory systems

## Abstract

Environmental awareness is an essential attribute of all organisms. The homeoviscous adaptation system of Bacillus subtilis provides a powerful experimental model for the investigation of stimulus detection and signaling mechanisms at the molecular level. These bacteria sense the order of membrane lipids with the transmembrane (TM) protein DesK, which has an N-terminal sensor domain and an intracellular catalytic effector domain. DesK exhibits autokinase activity as well as phosphotransferase and phosphatase activities toward a cognate response regulator, DesR, that controls the expression of an enzyme that remodels membrane fluidity when the temperature drops below ∼30°C. Membrane fluidity signals are transmitted from the DesK sensor domain to the effector domain via rotational movements of a connecting 2-helix coiled coil (2-HCC). Previous molecular dynamic simulations suggested important roles for TM prolines in transducing the initial signals of membrane fluidity status to the 2-HCC. Here, we report that individual replacement of prolines in DesKs TM1 and TM5 helices by alanine (DesKPA) locked DesK in a phosphatase-ON state, abrogating membrane fluidity responses. An unbiased mutagenic screen identified the L174P replacement in the internal side of the repeated heptad of the 2-HCC structure that alleviated the signaling defects of every transmembrane DesKPA substitution. Moreover, substitutions by proline in other internal positions of the 2-HCC reestablished the kinase-ON state of the DesKPA mutants. These results imply that TM prolines are essential for finely tuned signal generation by the N-terminal sensor helices, facilitating a conformational control by the metastable 2-HCC domain of the DesK signaling state.

## Abstract (precis)

Signal sensing and transduction is an essential biological process for cell adaptation and survival. Histidine kinases (HK) are the sensory proteins of two-component systems that control many bacterial responses to different stimuli, like environmental changes. Here, we focused on the HK DesK from Bacillus subtilis, a paradigmatic example of a transmembrane thermosensor suited to remodel membrane fluidity when the temperature drops below 30°C. DesK provides a tractable system for investigating the mechanism of transmembrane signaling, one of the majors interrogates in biology to date. Our studies demonstrate that transmembrane proline residues modulate the conformational switch of a 2-helix coiled-coil (2-HCC) structural motif that controls input-output in a variety of HK. Our results highlight the relevance of proline residues within sensor domains and could inspire investigations of their role in different signaling proteins.

## INTRODUCTION

Success in the biological world depends on the ability to sense and respond adaptively to environmental cues. Bacteria use extensively studied stimulus-response to cope with a variety of selective challenges: chemotactic signaling pathways that control locomotor behaviors and two-component signaling pathways that mediate changes in gene expression (1–3). Bacterial signaling systems offer powerful models for exploring molecular mechanisms of stimulus detection and response. The simplest two-component pathways comprise a transmembrane (TM) sensor kinase that detects an environmental stimulus and a cytoplasmic response regulator that produces an adaptive change in gene expression. Temporal changes in the properties of the TM helices of the sensor kinase are often a prerequisite for their functional activity (4–6). Over recent years, there has been a growing interest in describing the molecular mechanism of signal detection and transduction of these sensor proteins by means of structural and bioinformatics approaches (7–10). In spite of these efforts, the molecular nature of TM signaling in membrane-bound sensor kinases is still one of the central questions in bacterial physiology.

DesK is a TM histidine kinase (HK) which, together with its cognate response regulator, DesR, operates in Bacillus subtilis to control membrane lipid homeostasis. DesK is proposed to sense membrane thickness as a proxy for membrane viscosity and feedback onto lipid desaturation as a response to perturbation. Upon an increase in membrane viscosity by a temperature downshift, DesK phosphorylates the response regulator DesR, which induces transcription of the fatty acid desaturase Δ5-Des, which is encoded by the *des* gene. This DesK-dependent introduction of unsaturated fatty acid into the membrane enhances survival at low temperatures (11–13).

**FIG 1:**
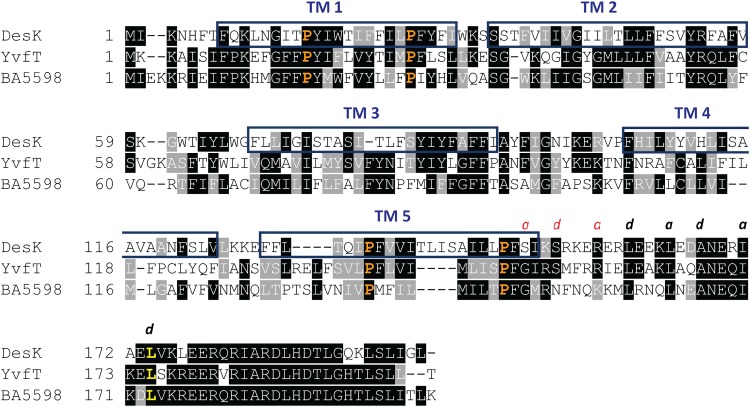
Alignment of the TM region of thermosensors homologous to DesK. Sequence alignment of the transmembrane helices (TM) and the 2-helix coiled coil (2-HCC) of B. subtilis DesK, YvfT, and BA5598 of B. anthracis was produced using T-Coffe software. Conserved proline residues studied in this work are shown in orange. Leu174 is highlighted in yellow. *a* and *d* indicate positions of the heptad repeats. The positions that stabilize the 2-HCC by hydrophobic interactions are in black.

DesK is a class I HK that works as a homodimer and, in contrast to most HKs, has no extracellular domains. Hence, the N-terminal sensor domain consists of ten TM helices, five coming from each protomer, arranged in an unknown fashion within the membrane. The fifth TM helix of each protomer (TM5) connects directly, without any of the intermediate domains found in other HKs (HAMP, PAS, or GAF), into a signaling helix, the 2-helix coiled coil (2-HCC), that ends up in the dimerization and histidine phosphotransfer domain (DHp), a 4-helix bundle (4-HB) (14). Systematic deletion of DesK’s TM segments and functional analysis showed that a truncated minimal sensor version, in which the N-terminal half of the first TM helix is fused to the C-terminal half of the fifth TM helix, dubbed MS-DesK, is fully functional both *in vitro* and *in vivo* (15). These studies highlighted that TM1 and TM5 have key structural elements for signal sensing and transduction. Several structural, computational, and functional studies on full-length DesK and MS-DesK showed that a stabilization/destabilization switch of the 2-HCC is crucial for the regulation of DesK activities (14, 16, 17). In the kinase-competent form, both helices are rotated by 90°, leading to 2-HCC unpacking. Therefore, the 2-HCC is expected to be stable in the phosphatase-competent state and disrupted in the kinase-competent state. The mechanism by which DesK detects environmental inputs at the sensor domain and propagates them, resulting in unwinding of the 2-HCC, is largely unknown. Toward a preliminary exploration of this mechanism, we performed simulations of each TM helix embedded in lipid membranes to test their sensitivity to membrane fluidity (16). These computational studies showed that while TM2, TM3, and TM4 present no large variations in conformational dynamics, TM1 and TM5 exhibit different tilting angles when simulated in membranes of different fluidity. The simulations further showed kinking of TM1 and TM5 at conserved proline (Pro) residues (Fig. 1) (16). These studies suggested that Pro-induced kinks on TM helices have an important role in transducing the initial signals to the cytoplasmic coiled coil. Importantly, the conservation of four Pro residues within the TM region of DesK sensors from B. subtilis and clinically relevant strains suggests an important functional role of these residues.

In this study, we show that when any of these Pro residues was mutated to alanine, in TM1 and TM5, the B. subtilis DesK output was shifted toward the kinase-OFF, phosphatase-ON state. Random amino acid mutagenesis of the sensor domain of the proline-to-alanine mutants (DesKPA) led to identification of a suppressor (L174P) that restored the signaling function of every DesKPA replacement. Remarkably, Leu174 is part of the hydrophobic core that stabilizes the 2-HCC in the phosphatase state, but it is rotated away and exposed to the solvent in the kinase conformation. Our study revealed evidence for a structural and signaling change mediated by prolines in TM1 and TM5 of DesK that might involve dynamic shifts over a range of sensor domain conformations that finely regulate input-output of the 2-HCC helices. This work provides molecular insight into the mechanistic basis of sense-and-respond systems for membrane-embedded sensor domains.

## RESULTS

### A site-directed mutagenesis approach uncovers functional significance of proline residues for DesK signaling.

**FIG 2:**
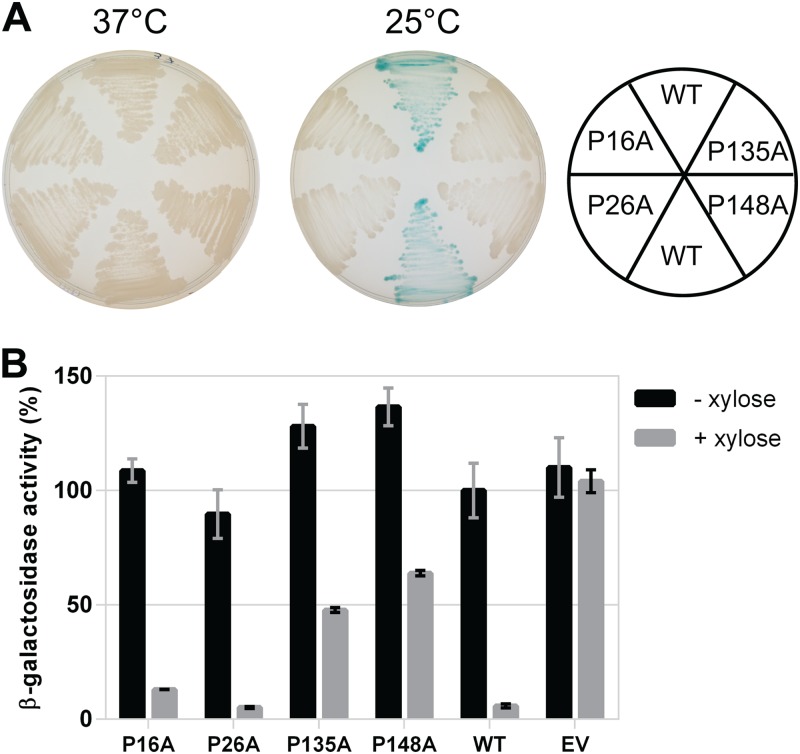
Pattern of P*des-lacZ* expression in the wild type and DesKPA mutants. Plasmids expressing different DesKPA mutants (DesK_P16A_, DesK_P26A_, DesK_P135A_, and DesK_P148A_) or wild-type DesK (WT) from the xylose-inducible promoter P*xyl*, as well as with empty vector (EV), were transformed in the reporter strains, as indicated. (A) DAK3 transformants were streaked in LB plates in the presence of 60 μg/ml X-Gal and 0.05% xylose and incubated at 37°C (left) or for 5 h at 37°C and then transferred to 25°C for 24 h (right). (B) AKP20 transformants were grown in LB at 37°C in the absence (black bars) or presence (gray bars) of 0.05% xylose. Shown values correspond to the β-galactosidase activity of aliquots taken after 6 h of growth, expressed as a percentage of wild-type activity in the absence of xylose.

Sequence alignments as well as molecular modeling and simulations suggested that DesK’s TM Pro residues, particularly Pro16 in TM1 and Pro148 in TM5, are important for signal sensing and/or transduction (16, 17). Moreover, sequence analysis of DesK homologs of B. subtilis and B. anthracis, which also respond to temperature changes (data not shown), revealed the conservation of a total of four Pro residues located within their TM segments (Fig. 1). Of these, Pro16 and Pro26 belong to TM1, while Pro135 and Pro148 belong to TM5. In order to explore the contribution of each of these Pro residues to DesK’s TM signaling mechanism, we constructed site-directed mutants with single-point Pro-to-Ala replacements (here designated DesKPA mutants). To assess the effects of these substitutions on DesK kinase activity, we engineered a DesK-less host containing the β-galactosidase reporter gene fused to the desaturase promoter (P*des-lacZ*) (17). In this strain, named DAK3 (Table 1), the levels of β-galactosidase activities rely on activation of P*des*, which ultimately depends on the flux of phosphate from DesK to DesR. The induction of the P*des-lacZ* fusion by the DesK variants expressed in this strain were easily monitored on solid medium containing X-Gal (5-bromo-4-chloro-3-indolyl-β-d-galactopyranoside), where the colonies turn blue if the HK is in a kinase-ON state and remain white if the HK is in a kinase-OFF state (13). As shown in Fig. 2A, expression of wild-type (WT) DesK from a xylose-inducible promoter stimulates transcription of the reporter gene when cells are incubated at low temperature (25°C), rendering blue colonies. In contrast, at 37°C the colonies remain white. We observed that the amino acid substitutions P16A, P26A, P135A, and P148A shift the sensor to a state unable to stimulate *des* transcription at 25°C (Fig. 2A). Subsequently, we tested whether these DesK variants display *in vivo* phosphatase activity using strain AKP20 (Table 1). This strain is a DesK-less host that exhibits constitutive expression of P*des* and, hence, high β-galactosidase activity levels due to DesR overexpression from the strong constitutive kanamycin promoter (P*km*) and its phosphorylation from small phosphordonors or other kinases (13). Expression in AKP20 of WT DesK or DesK variants that exhibit DesR∼P phosphatase activity is recorded as a reduction in the β-galactosidase activity encoded by P*des-lacZ* (13, 17). As shown in Fig. 2B, the DesK_P16A_ and DesK_P26A_ mutants show β-galactosidase activities similar to that of the WT DesK, indicating that they are capable of dephosphorylating DesR. The variants DesK_P135A_ and DesK_P148A_ also show reduction of β-galactosidase activities, although to a lesser extent than the WT sensor. Taken together, these *in vivo* results show that all single Pro-to-Ala replacements in DesK TM segments lock the sensor in a phosphatase-ON/kinase-OFF state.

**TABLE 1:** Strains and plasmids used in this study

Strain or plasmid	Relevant characteristic(s)	Source or reference
Strain		
Escherichia coli		
DH5α	*supE44 thi-1 ΔlacU169* (Φ80*lacZ*ΔM15) *endA1 recA1 hsdR17 gyrA96 relA1 trp-6 cysT329*::*lac inm^pI(209)^*	Laboratory stock
Bacillus subtilis		
JH642	*trpC2 pheA1*	Laboratory stock
DAK3	JH642 *amyE*::P*des-lacZ desK*::*km* P*xyl-desR*; Cm^r^ Km^r^	17
AKP20	JH642 *amyE*::P*des-lacZ desK*::*km* P*km-desR*; Cm^r^ Km^r^	13
Plasmid		
pHPKS	B. subtilis replicative vector of low copy number, Erm^r^ Lm^r^	3
pARD7	pHPKS derivative with P*xyl* promoter cloned in SacI	35
pARD8	pHPKS derivative with P*xyl* promoter cloned in SacI and *desKC* end in SmaI/PstI	35

### Second-site suppression of DesKPA signaling defects.

To further characterize the DesKPA mutants, we isolated suppressor mutations that restored their kinase activity at 25°C, expecting that suppressor analysis might reveal the role of TM Pro residues in signaling. To this end, we performed random mutagenesis of each of the *desKPA* alleles, specifically on the nucleotide region coding for the sensor domain and part of the 2-HCC. We screened this mutant library in Luria-Bertani (LB) plates containing X-Gal and selected the clones that formed blue colonies at 25°C and white colonies at 37°C, indicating restoration of the kinase activity at low temperatures and phosphatase activity at high temperatures. Using this screening procedure, we isolated the double mutants DesK_P135A-L174P_ and DesK_P148A-L174P_, both containing the same suppressor mutation, L174P. Leu174 is part of DesK’s 2-HCC and resides in a *d* position of the amino acid heptad repeat *abcdefg* (14, 17). In 2-HCCs, the interaction is sustained by a hydrophobic core fixed by residues *a* and *d* from each α-helix, which conform a stabilizing seal (Fig. 1) (18).

**FIG 3:**
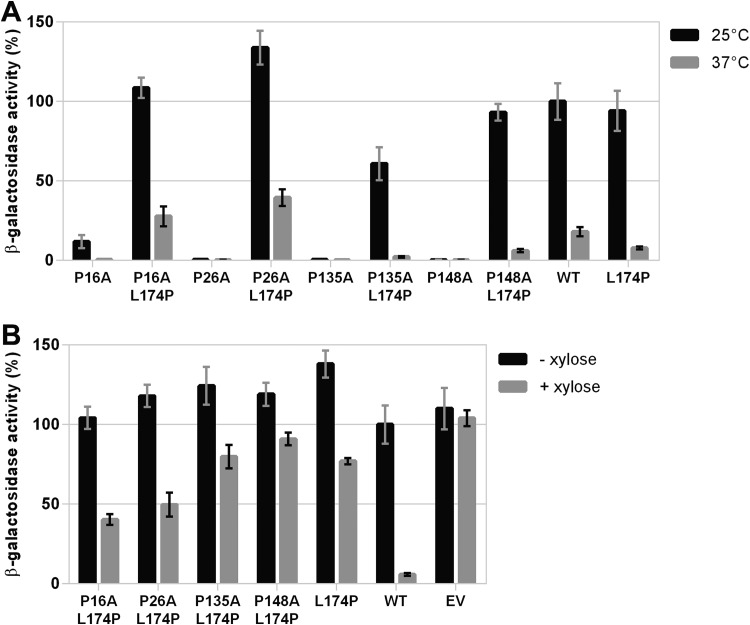
DesKPA_L174P_ double mutants respond to temperature. Plasmids expressing each of the DesKPA_L174P_ double mutants (DesK_P16A-L174P_, DesK_P26A-L174P_, DesK_P135A-L174P_, and DesK_P148A-L174P_), the DesKPA single mutants (DesK_P16A_, DesK_P26A_, DesK_P135A_, and DesK_P148A_), DesK_L174P_, or wild-type (WT) DesK from the xylose-inducible promoter P*xyl*, as well as with the empty vector (EV), were transformed in the reporter strains as indicated. (A) DAK3 transformants were grown in LB in the presence of 0.05% xylose at 37°C. At an OD_525_ of 0.3 the cultures were divided into two fractions. One of them was transferred to 25°C (black bars), and the other one was kept at 37°C (gray bars). Shown values correspond to the β-galactosidase activity of aliquots measured 4 h after cold shock, expressed as a percentage of wild-type activity at 25°C. (B) AKP20 transformants were grown in LB at 37°C in the absence (black bars) or presence (gray bars) of 0.05% xylose. Shown values correspond to the β-galactosidase activity of aliquots measured after 6 h of growth, expressed as a percentage of wild-type activity in the absence of xylose.

Since the substitution L174P reestablished the thermosensing ability of two different DesKPA variants, we tested if this mutation also suppresses the kinase-OFF state of DesK_P16A_ and DesK_P26A_. Notably, the double mutants DesK_P16A-L174P_ and DesK_P26A-L174P_ behaved similarly to WT DesK, i.e., inducing the expression of P*des* at 25°C and repressing it at 37°C (Fig. 3A). We also found that DesK_P16A-L174P_, DesK_P26A-L174P_, DesK_P135A-L174P_, and DesK_P148A-L174P_ are capable of dephosphorylating DesR~P, although less efficiently than the corresponding DesKPA single mutants (Fig. 3B). These experiments show that the L174P mutation reestablishes the cold-induced kinase activity of the DesKPA variants, but their phosphatase activities are diminished at 37°C (8- to 15-fold less β-galactosidase activity than WT DesK) (Fig. 3B).

### Second-site suppression of DesK kinase-OFF mutants.

**FIG 4:**
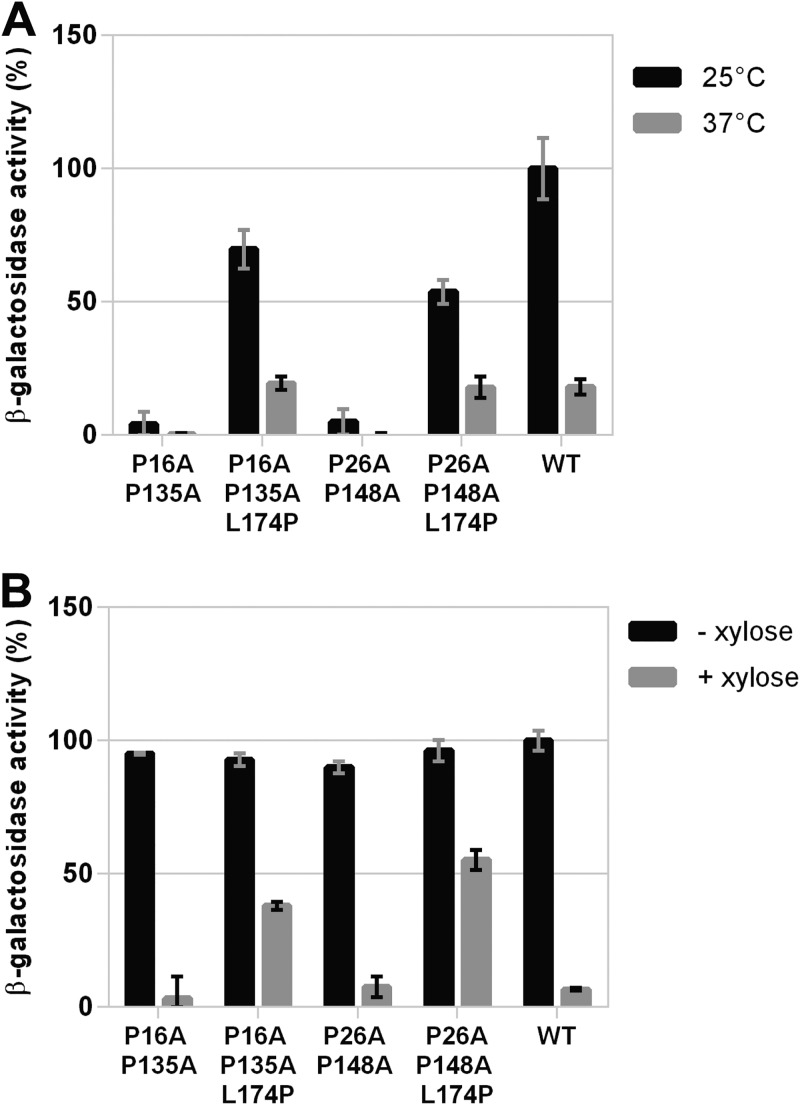
Effect of L174P replacement on DesKPA variants containing two Pro-to-Ala mutations. (A) Kinase activity assay of DAK3 cells expressing the indicated DesK variants that were grown in LB in the presence of 0.05% xylose at 37°C. At an OD_525_ of 0.3, the cultures were divided into two fractions. One of them was transferred to 25°C (black bars), and the other one was kept at 37°C (gray bars). Shown values correspond to the β-galactosidase activity of aliquots measured 4 h after cold shock, expressed as a percentage of wild-type activity at 25°C. (B) Phosphatase activity assay of AKP20 cells expressing the indicated DesK variants grown in LB at 37°C in the absence (black bars) or presence (gray bars) of 0.05% xylose. Shown values correspond to the β-galactosidase activity of aliquots taken after 6 h of growth, expressed as a percentage of wild-type activity in the absence of xylose.

**FIG 5:**
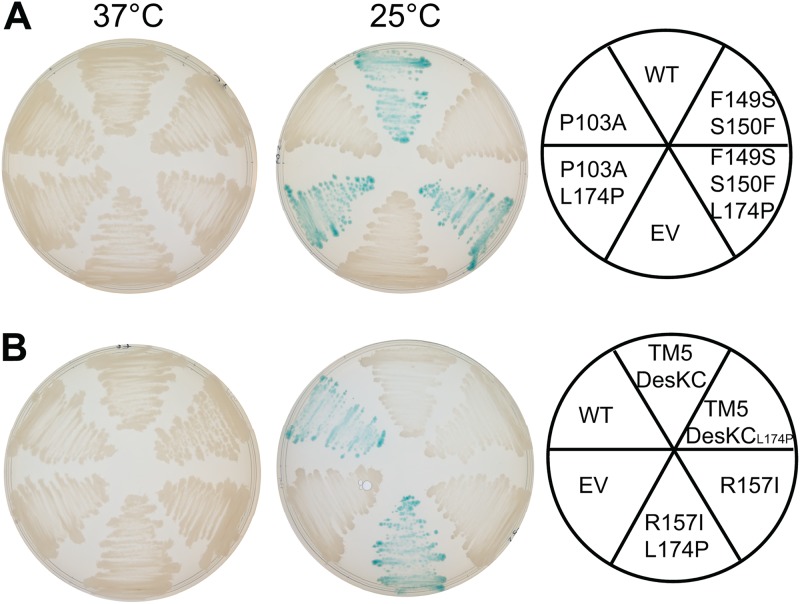
Pattern of P*des-lacZ* expression in different DesK variants. Strain DAK3 was transformed with plasmids expressing wild-type DesK (WT) or different DesK alleles under the control of the xylose-inducible promoter P*xyl*, as well as with the empty vector (EV). The resulting strains were streaked in LB plates in the presence of 60 μg/ml X-Gal and 0.05% xylose and incubated at 37°C (left) or for 5 h at 37°C and then transferred to 25°C for 24 h (right).

To gain insight into the nature of the effect caused by L174P, we explored whether this mutation could functionally suppress the signaling defect of distinct DesK mutants. We found that L174P suppresses the kinase-OFF output caused by double Pro-to-Ala replacements in both TM1 and TM5 (DesK_P16A-P135A_ and DesK_P26A-P148A_) (Fig. 4A), although the phosphatase activity of the complemented mutants was reduced between 8- and 12-fold (Fig. 4B). We also found that L174P restores the temperature-regulated kinase activity of the R157I substitution that locks DesK in a phosphatase-ON state by stabilization of the 2-HCC region (Fig. 5). In addition, we determined that L174P is able to abrogate the kinase-OFF state of DesK_F149S-S150F_ and DesK_P103A_ mutants (Fig. 5). Altogether, these results show that L174P reestablishes the functionality of sensors with amino acid replacements in either the sensor or the 2-HCC domains, probably through compensatory conformational changes that restore the sensory adaptation of the different mutants.

The finding that L174P suppresses the kinase-OFF state of mutants blocked in either signal sensing or transduction, combined with the fact that residue 174 occupies a *d* position within the 2-HCC, raised the possibility that this modified domain containing a Pro in position 174 was working as a temperature sensor *per se*. To test this possibility, we used a chimera where the fifth TM segment (TM5) of DesK is directly connected to the catalytic core (DesKC) via the 2-HCC. This fusion (TM5-DesKC) is devoid of kinase activity at either 25 or 37°C (15). Figure 5 shows that TM5-DesKC_L174P_ is still unresponsive to a low-temperature signal. We conclude that the modified 2-HCC is not a temperature viscosity sensor by itself, implying that additional elements of the TM region are important for signal detection and transduction.

### Proline-mediated destabilization of the 2-HCC restores the signaling defect of a DesKPA mutant.

**FIG 6:**
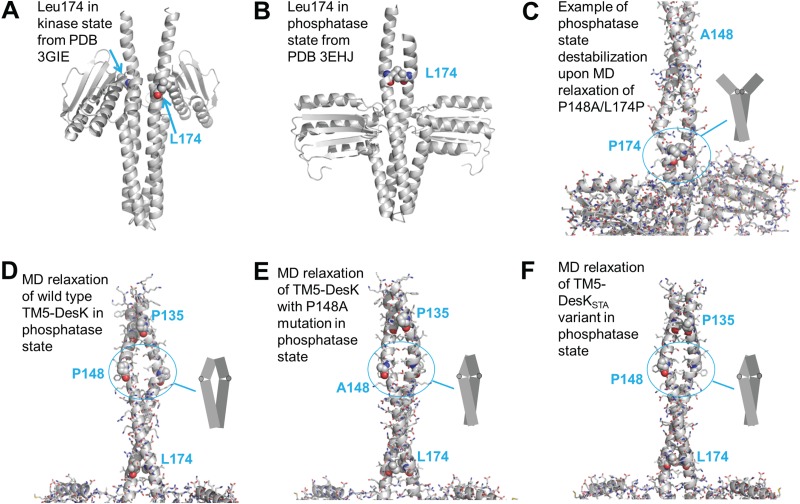
Destabilizing effect of Pro174. (A and B) X-ray structure of DesKC in kinase-like (PDB entry 3GIE) (A) and phosphatase (PDB entry 3EHJ) (B) states, with Leu174 rendered as spheres to highlight its location in the 2-HCC. (C) Instability introduced by mutation L174P on the 2-HCC in a model of TM5-DesKC-P148A/L174P in the phosphatase state; notice the breaks just upstream of Pro174. (D) Hydrated state observed in an molecular dynamics simulation of wild-type TM5-DesKC in the phosphatase state; notice the slight opening around Pro148 (from our previous work [17]). (E) Compact state as observed upon molecular dynamics simulation of TM5-DesKC with only a P148A substitution; notice the more compact 2-HCC and compare to that of TM5-DesKC_P148A-L174P_ in panel C and in Fig. S1 at https://doi.org/10.5281/zenodo.3523363. (F) Compact state as observed upon molecular dynamics simulation of TM5-DesKC with 3 substitutions that stabilize the 2-HCC in the phosphatase state (DesK_STA_ from our previous work [17]). Notice an effect similar to that seen in panel E.

Given the ability of the L174P substitution to restore the response to low temperature of different DesK sensor domain mutant versions, we focused on the structural aspects related to this modification. Leu174 is resolved in X-ray structures of DesKC mimicking the phosphatase- and kinase-competent forms (14). This residue is part of the hydrophobic core that stabilizes the 2-HCC in the phosphatase state but is rotated away and exposed to the solvent in the kinase conformation (Fig. 6A and B) (14, 17). MD simulations of the double mutant TM5-DesK_P148A-L174P_ show that Pro174 clearly exerts a strong destabilizing effect in a model built from the structure of the phosphatase state (compare representative conformations from Fig. 6C and E with additional data in Fig. S1 in the supplemental material at https://doi.org/10.5281/zenodo.3523363). This is because, as mentioned above, residue 174 lies at the internal side of the 2-HCC in this conformation, so the kink introduced by the Pro pushes the helices apart, destabilizing it (Fig. 6C). This could explain why variants with the L174P mutation exhibit lower phosphatase activity than WT DesK: such mutants cannot stabilize a phosphatase conformation as efficiently as the WT protein due to perturbation of the 2-HCC. In contrast, position 174 is rotated away in the kinase conformation (Fig. 6A) and the 2-HCC is intrinsically destabilized, as we previously showed (17); i.e., position 174 is no longer internal and, moreover, there is no 2-HCC that can be disrupted by the L174P substitution. Thus, this mutation is expected to be much more destabilizing in the phosphatase than in the kinase form.

**FIG 7:**
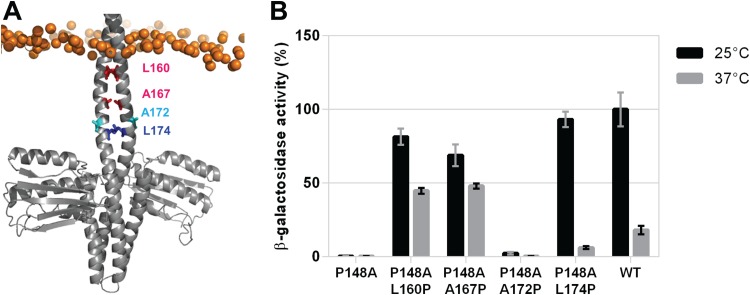
Proline insertions at internal sites of 2-HCC also restore kinase activity of DesK_P148A._ (A) DesK’s DHp domain and 2-HCC linker in the phosphatase state (PDB entry 3EHJ). Mutagenized residues are highlighted: pink spheres represent those amino acids occupying *d* positions, and, thus, participating in the stabilization of the 2-HCC, as L174 (blue spheres); cyan spheres represent residues located on the opposite side of the helix. (B) β-Galactosidase activity of strain DAK3 transformed with plasmids expressing wild-type DesK (WT), DesK_P148A_, DesK_P148A-L160P_, DesK_P148A-A167P,_ DesK_P148A-A172P_, or DesR_P148A-L174P_ from the xylose-inducible promoter P*xyl*. Strains were grown in LB in the presence of 0.05% xylose at 37°C. At an OD_525_ of 0.3, the cultures were divided into two fractions. One of them was transferred to 25°C (black bars), and the other one was kept at 37°C (gray bars). Shown values correspond to the β-galactosidase activity of aliquots measured 4 h after cold shock, expressed as a percentage of wild-type activity at 25°C.

Taking this into account, we decided to analyze if substitutions by Pro at other internal positions of the 2-HCC (i.e., in register with L174) can also recover the kinase activity of the DesKPA mutants. Thus, we generated DesK_P148A-L160P_ and DesK_P148A-A167P_ double mutants, since L160 and A167 occupy *d* positions of the heptad repeats of the 2-HCC, like L174 (Fig. 1 and 7A). The *in vivo* kinase activity assays for both mutant proteins showed that they have the ability to activate P*des* transcription in a constitutive manner, that is, a kinase-ON state was observed at both 25°C and 37°C (Fig. 7B). On the other hand, we constructed the double mutant DesK_P148A-A172P_, where residue A172 occupies a *b* position of the 2-HCC, implying that it is located in the opposite side of the helix relative to residues at *d* positions (i.e., outer face of the 2-HCC; Fig. 7A) and, hence, does not participate in the stabilization of the 2-HCC. We expected that the introduction of a Pro residue at this position would not disrupt the 2-HCC; therefore, the kinase-OFF output of the DesK_P148A_ mutant would not be suppressed. As predicted, DesK_P148A-A172P_ is unable to induce *des* expression in strain DAK3 (Fig. 7B). These results indicate that the kinks introduced in stabilizing positions of the 2-HCC (P160, P167, or P174) disrupt its hydrophobic core, favoring the kinase state of the protein and therefore recovering the kinase activation capacity lost in the insensitive DesK_P148A_ mutant. However, the effect of the disruption in DesK_P148A-L160P_ and DesK_P148A-A167P_, where the Pro substitution is closer to the TM sensor domain than in DesK_P148A-L174P_, seems to be more dramatic, leading to a constitutive kinase-ON state of the sensor.

**FIG 8:**
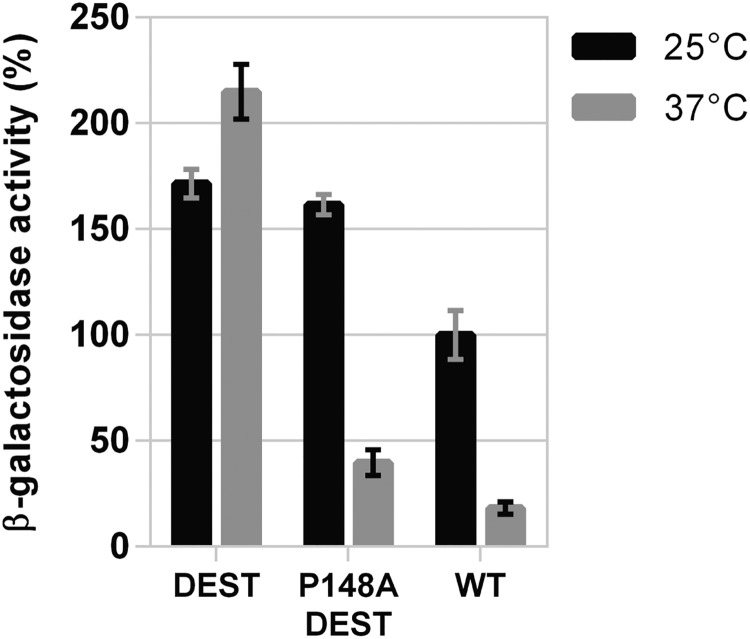
Effect of 2-HCC destabilization on DesK_P148A_ mutant. β-Galactosidase activity of strain DAK3 transformed with plasmids expressing wild-type DesK (WT) or DesK_DEST_ or DesK_P148A-DEST_, from the xylose-inducible promoter P*xyl*. Strains were grown in LB in the presence of 0.05% xylose at 37°C. At an OD_525_ of 0.3, the cultures were divided into two fractions. One of them was transferred to 25°C (black bars), and the other one was kept at 37°C (gray bars). Shown values correspond to the β-galactosidase activity of aliquots measured 4 h after cold shock, expressed as a percentage of wild-type activity at 25°C.

As shown above, we found that destabilization of DesK’s 2-HCC by introduction of a Pro residue that disrupts the hydrophobic core shifted the kinase-OFF output of DesK_P148A_ toward the kinase-ON state. To confirm that destabilization of the 2-HCC by amino acid substitutions other than proline is able to revert the kinase-OFF state of DesK_P148A_, we resorted to the previously characterized DesK_DEST_ mutant. This variant possesses constitutive and exacerbated kinase activity due to the destabilization of the 2-HCC by replacement of three hydrophobic amino acids occupying *a* and *d* positions of its heptad repeats with polar charged and uncharged residues (A167R, I171G, and L174G) (17). We generated the DesK_P148A-DEST_ mutant (DesK_P148A-A167R-I171G-L174G_), and its kinase activity was evaluated *in vivo* in strain DAK3 at 25 and 37°C. Consistent with the idea that any amino acid substitutions that weaken the formation of the 2-HCC would be expected to shift DesK_P148A_ from a kinase-OFF to a kinase-ON state, we found that DesK_P148A-DEST_ displays a high β-galactosidase activity at 25°C as a result of its high kinase activity (Fig. 8). Moreover, DesK_P148A-DEST_ output activity is temperature regulated (Fig. 8).

### Suppression of MS-DesK_P148A_ mutant.

**FIG 9:**
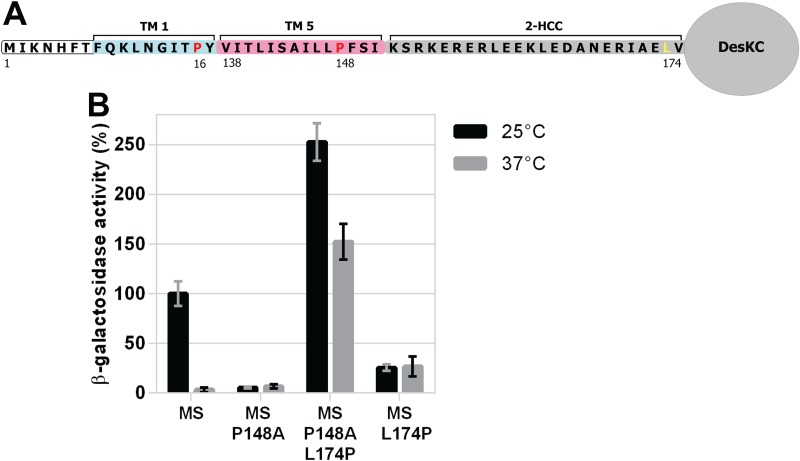
(A) Representation of MS-DesK minimal sensor. The extracellular N-terminal region of DesK is depicted in white. The single chimeric TM segment of MS-DesK is composed of the first 10 residues of DesK’s TM helix 1 (light blue) fused to the last 14 residues of TM helix 5 (pink). The adjacent 2-HCC and catalytic DesKC domain are shown in gray. The conserved prolines Pro16 and Pro148 are highlighted in red and Leu174 in yellow. (B) Effect of P148A and L174P mutations on DesK minimal sensor kinase activity. Strain DAK3 was transformed with plasmids expressing different MS-DesK alleles (MS, MS_P148A_, MS_P148A-L174P_, and MS_L174P_) under a xylose-inducible promoter. The resulting strains were grown in LB in the presence of 0.05% xylose at 37°C. At an OD_525_ of 0.3 the cultures were divided into two fractions. One of them was transferred to 25°C (black bars), and the other one was kept at 37°C (gray bars). Shown values correspond to the β-galactosidase activity of aliquots measured 4 h after cold shock, expressed as a percentage of MS-DesK activity at 25°C.

The engineered minimal sensor MS-DesK retains wild-type sensing and transmission capacity *in vivo* and *in vitro*. In this construct, the first 17 residues of the N-terminal domain of DesK are linked to the last 14 residues of the C-terminus of TM5 (Fig. 9A). In agreement with a recent report (19), we found that the substitution P148A in MS-DesK causes a kinase-OFF output (Fig. 9B). To test whether the suppressor L174P shifts MS-DesK toward the kinase-ON state, we constructed the double mutant MS-DesK_P148A-L174P_ (full-length DesK numbering). The β-galactosidase activity of this variant was 2.5 times higher at 25°C than the activity of the parental protein MS-DesK but also, unexpectedly, 150 times higher at 37°C (Fig. 9B). These results imply that this double mutation locked the minimal sensor into an exacerbated kinase-ON output. This finding differs from that of full-length DesK_P148A-L174P_ in that its kinase activity is almost similar to that exhibited by WT DesK at each temperature (Fig. 3). It is likely that proline-mediated disruption of the 2-HCC in the simplified MS-DesK sensor is generating more drastic rearrangements, in the delicate balance of protein conformation and communication between subdomains, than in its full-length progenitor DesK. Evidently the artificially construct MS-DesK does not retain all the properties of its intact progenitor, but the results shown in Fig. 9B indicate that suppression-dependent output control by L174P still operates in MS-DesK_P148A_ despite the upregulated kinase activity of the resulting minisensor.

## DISCUSSION

HKs have been studied for decades, yet there is no clear understanding about how they generate and transmit signals from the sensor to the downstream effector-catalytic domain. Over the last few decades, our group has been studying DesK, a relatively simple HK without extracytoplasmic sensor domains and lacking any linker HAMP, PAS, or GAF domain. We have established that DesK’s signal transduction mechanism should lead to rotation of the DHp helices to control DesK output activity, and that the activation switch is under the control of a 2-HCC connecting sensor and effector domains (14, 17). Initial signal generation is the least clear aspect of the mechanism underlying DesK’s functioning and of HK mechanisms in general. However, decades of work on HKs suggest initial sensing exerts on the TM helices combinations of helical rotation, tilting, and piston-like motions, possibly different for each system but all ultimately regulating stabilization/destabilization of the 2-HCC and induction of asymmetric states upon activation (20–24). In DesK, sensing *per se* and transmission to the downstream domains must both be in charge of the TM domain.

Our previous work using atomistic molecular dynamics simulations suggested that DesK’s TM helices 1 and 5 are mechanically sensitive to membrane status, specifically, that membrane fluidity could affect the orientation and vertical positioning of the helices thanks to pivoting of helices around the discontinuities induced by the proline residues, especially P16 in TM helix 1 and P148 in TM helix 5. More globally, prolines have been confirmed to have functional mechanical roles in a number of systems, often by introducing flexibility in helices and most notably when part of GXXP motifs, as in DesK’s Pro16 (25–28). In the current work, we have performed site-directed mutagenesis on the four conserved Pro residues located in DesK’s TM domain in order to investigate the TM signaling mechanism of DesK. All four Pro residues proved to be essential to trigger DesK kinase activity, as their mutation to alanine disrupted activation. Prolines 16, 26, 135, and 148 are clearly located within TM helices, the former two in TM helix 1 and the latter two in TM helix 5. In these sites, alterations to alanine are expected to restore helical continuity, disrupt hinges, and abolish flexibility; thus, we interpret our findings as evidence that the proline-induced hinges are important for signal sensing and signal transduction in DesK. Considering our previous simulation results, the signal generated by DesK’s TM domain could consist of helical displacements along the membrane normal facilitated by pivoting around the prolines, accompanied by variable TM helix tilting. On the other hand, P103, which is located in the sensor domain but in an interhelical loop (right upstream of TM helix 4), turned out also to be essential to activate DesK kinase activity (Fig. 5). In this case it is difficult to draw conclusions about its mechanistic contribution, but it might have a specific role, since a Pro residue is also present in the TM3-TM4 interhelical loop of the homologous thermosensors, although its position conservation is lower than that of the other four TM proline residues.

Among prolines 16, 26, 135, and 148, the former and the latter are the most highly conserved ones in large alignments (16). They are also the ones embedded deepest in the membrane, close to the center of the helices that contain them: Pro16 is 8 residues away from the N-terminal end of TM helix 1 (F8 is the first helix residue), whereas Pro26 is only 4 residues away from its C-terminal end. This difference is even more pronounced if TM helix 1 actually extends further upstream, as some secondary structure predictions suggest. In turn, P135 is 6 residues away from the N-terminus of TM5, while P148 is preceded and followed by a large helical section both upstream in TM helix 5 and downstream into the 2-HCC that presumably continues smoothly but with reversed polarity, as suggested by secondary structure, TM, and coiled-coil predictions (17). Given these observations, P16 and P148 might be the most important proline residues for the activation mechanism, a proposal consistent with the MS-DesK construct (which only has these two TM prolines) being cold sensitive. However, our results show that all prolines and all TM helices are required to achieve robust fine-tuning of the activity, which could explain why MS-DesK is not a natural temperature sensor.

If P16 and P148 are in fact the key elements for signal generation and transduction, what might their roles be? Whereas full experimental structures are required to make more solid hypotheses, we can advance a scenario based on secondary structure predictions, partial models, our previous experimental and simulation data, and our new results on the proline mutants. The N-terminus of TM helix 1 contains the “sunken buoy” motif, reported to be sensitive to membrane thickness variations (15). This motif features a set of polar residues that would prefer to stay at the aqueous surface when membrane permits. The highly conserved pair G13-P16 could provide a very flexible hinge, as observed in our previous simulations on this TM helix (16, 17) and in several other systems (29–32). Such a hinge could enable regulation of the exact angle between both helix segments around P16. In a fluid membrane context, the hinge would allow for exposure of the polar residues of the first helical segment adopting a position nearly perpendicular to the membrane normal, leaving the second half (i.e., after P16) parallel to this vector, i.e., forming an angle close to 90 degrees between them. Upon a fluidity decrease and the concomitant membrane thickening (15, 17, 19), the first helical segment would move around the hinge, becoming almost parallel to the membrane normal, allowing the polar residues to snorkel, looking for a polar environment. This movement would result in up-down motion of TM helix 1 along the membrane normal. According to our previous simulations, TM helix 5 is also sensitive to membrane status, so its disposition within the membrane is also expected to vary upon a change in the plasma membrane properties by pivoting on P148, which could cause an up and down (i.e., piston-like) movement of the second half of TM helix 5 (16). Under such a scenario, it is possible that an extensive reorganization of DesK’s TM domain will be triggered upon stimulation. In addition, our results from this and previous work clearly indicate that the bulge created by P148 at the end of the TM domain (TM helix 5) interplays with the 2-HCC element. P148 generates a polar cavity that allows hydration and opening of the 2-HCC (Fig. 6D and reference 17). Replacement of P148 by alanine favors sealing of this polar opening and also relaxation of the bulge it provokes into a smooth coiled-coil structure (Fig. 6E), an effect similar to that displayed by the mutations to hydrophobic amino acids in DesK_STA_ from Saita and coworkers (17) (Fig. 6F). Indeed, the stabilizing P148A mutation, which hampers DesK activation, presumably by sealing the bulge (Fig. 6C), can be compensated for by the DEST set of mutations that destabilizes the 2-HCC (17) (Fig. 8). Notably, Pro148 is in a position similar to that of NarQ’s P179, which is part of its HAMP domain and was suggested to be in charge of converting piston-like displacements coming from the sensor through the transmembrane domain into helical rotations to be fed into the DHp domain (see Fig. S2 at https://doi.org/10.5281/zenodo.3523363) (5). If this similar location reflects a similar functional role, P148 could be DesK’s natural decoder of piston-like displacements of the TM5 helices into helical rotations that promote 2-HCC destabilization and opening upon stimulation. This way, although DesK lacks a formal HAMP domain, its function would be built into the TM domain itself, through P148. The decoding function of P148 is possibly permitted thanks to the presence of the highly charged segment just C-terminal to the TM region (K_152_SRKERERLEEK_163_). This stretch of charged amino acids is anchored to the cytoplasmic polar side of the membrane, limiting vertical movements of the 2-HCC helices when the membrane properties change but allowing them to rotate (17).

In this work, we further found the L174P replacement as a suppressor mutation that restores the ability of every proline-to-alanine mutant to respond to cold. This suppressor worked not only for all the DesKPA alleles but also for a variety of mutants in the TM domain, indicating a general capacity of P174 to reconvert DesK-insensitive variants into cold sensors. Again, this effect seems to be mediated by regulation of 2-HCC stability, as the kink that P174 introduces at the internal side of the 2-HCC pushes the helices apart, destabilizing it and favoring the kinase conformation of the sensor (Fig. 6C). Introducing proline residues at other internal positions of the 2-HCC (residues 160 and 167) also restores kinase output but in an unregulated manner.

In summary, we (i) highlight once more the fine balance between 2-HCC stability and functional output, (ii) show that TM prolines are essential for finely tuned signal generation, and (iii) suggest that P148 is required to convert piston-like motions from the TM domain into the helical rotations that activate the 2-HCC.

## MATERIALS AND METHODS

### Bacterial strains and growth conditions.

Bacterial strains and plasmids used in the present study are listed in Table 1. Escherichia coli and Bacillus subtilis strains were routinely grown in Luria-Bertani (LB) broth at 37°C. Antibiotics were added to media at the following concentrations: erythromycin (Erm), 0.5 μg ml^−1^; lincomycin (Lm), 12.5 μg ml^−1^; chloramphenicol (Cm), 5 μg ml^−1^; kanamycin (Km), 5 μg ml^−1^; ampicillin (Amp), 100 μg ml^−1^. For the experiments involving expression of DesK variants under the control of the inducible P*xyl* promoter, 0.05% xylose was added (33).

### Plasmid and strain constructions.

All the mutants employed in this study were constructed by site-directed mutagenesis using overlap extension PCR. DNA fragments were obtained using the mutagenic oligonucleotides listed in Table 2, with DesK_WT_ or MS-DesK as the template. The open reading frames (ORF) coding for each mutant were finally amplified using oligonucleotides DesKBamHI33 Up and DesKPst Lw (Table 2, restriction sites underlined). Each mutant allele was cloned into the BamHI and PstI sites of the replicative plasmid pARD7, a derivative of the pHPKS vector (34) that allows expression of the genes under the control of P*xyl* (35). Finally, the plasmids were used to transform DAK3 (*desK*::*km* P*xyl-desR amyE*::P*des-lacZ*) (17) and AKP20 (*desK*::*km* P*km-desR amyE*::P*des-lacZ*) (13).

**TABLE 2:** Oligonucleotides used in this study

Name	Sequence^*a*^
DesKBamHI33Up	AGTAACATGGATCCCAGAAAATGAGGTAAGATC
DesKPstILw	GCTGATCTTCTGCAGTAAATATACTAATC
DesKSmaLw	GGAGATCCCGGGCAATTCGCTGACG
Mutagenic oligonucleotides	
P16A Up	TAAACGGGATTACGGCTTATATATGGAC
P16A Lw	CATATATAAGCCGTAATCCCGTTTAG
P26A Up	ATTTTTCATCCTCGCTTTCTACTTTATA
P26A Lw	AAGTAGAAAGCGAGGATGAAAAATATC
P135A Up	AAATTGCTTTTGTCGTTATTACCCTCAT
P135A Lw	AAAAGCAATTTGTGTCAGAAAGAATTC
P148A Up	CGCAATTTTATTGGCTTTCAGTATAA
P148A Lw	TTATACTGAAAGCCAATAAAATTGC
P103A Up	ATTAAGGAACGCGTCGCTTTTCATATT
P103A Lw	ATAAAATATGAAAAGCGACGCGTTCCTTAAT
L174P Up	GGATTGCAGAACCGGTAAAATTAGAAG
L174P Lw	TTCTTCTAATTTTACCGGTTCTGCAATCC
A172P Up	ATGAACGGATTCCAGAACTGGT
A172P Lw	TTACCAGTTCTGGAATCCGTTC
A167P Up	AGCTCGAGGATCCAAATGAACGG
A167P Lw	ATCCGTTCATTTGGATCCTCGAGC
L160P Up	GCGCGAACGACCTGAAGAAAAGC
L160P Lw	TCGAGCTTTTCTTCAGGTCGTTCGC
S149 F150 Up	TTTATTGCCCAGTTTCATAAAAAGCCG
S149 F150 Lw	GGCTTTTTATGAAACTGGGCAATAAAATTG

a Underlining indicates restriction sites.

### Mutagenesis by error-prone PCR.

Error-prone PCR was carried out over the *desKPA* alleles *desK_P16A_*, *desK_P26A_*, *desK_P135A_*, and *desK_P148A_*. DesKBamHI33UP and DesKSmaLw where used to amplify 560 nucleotides of the N-terminal region of DesK, containing the transmembrane segment plus 27 amino acids. The reaction mixture contains 1× *Taq* reaction buffer, 3.2 μM each oligonucleotide, 200 μM dATP, dGTP, and dTTP, 1 mM dCTP, 7 mM MnCl_2_, 0.15 mM MgCl_2_, and *Taq* DNA polymerase without proofreading activity. The mixture was heated at 94°C for 2 min, followed by 19 cycles of incubation at 94°C for 30 s, 57°C for 45 s, and 72°C for 1 min. The *desKPAtms* fragments obtained where digested with BamHI and SmaI and cloned into pARD8 (35). This plasmid is a pARD7 derivative that allows cloning the N-terminal *desK* end upstream of the wild-type *desKC* end, regenerating full-length *desK* sequence under the control of the P*xyl* promoter. Ligation mixtures where transformed into E. coli DH5α. The recombinant plasmids of a pool of transformants were extracted using the Promega Wizard Plus SV miniprep DNA purification system and then transformed into B. subtilis DAK3.

### β-Galactosidase activity assays.

B. subtilis *desK* mutant cells harboring a P*des*-*lacZ* transcriptomic fusion (DAK3; *desK*::*km-*P*xyl-desR amyE*::P*des-lacZ*) were transformed either with pHPKS empty vector, pHPKS/P*xyl-desKwt*, or pHPKS expressing different DesK variants under the inducible P*xyl* promoter. The resulting strains were grown overnight in LB in the absence of xylose and then diluted in the same medium to a final optical density at 525 nm (OD_525_) of 0.10. The inductor xylose then was added, and the cultures were grown at 37°C with shaking. At an OD_525_ of 0.3, the cultures were divided into two fractions, and one of them was transferred to 25°C and the other one was kept at 37°C. After each treatment, samples were taken at 1-h intervals and assayed for β-galactosidase activity as previously described (36). Briefly, cells were pelleted, diluted in Z buffer, and disrupted using lysozyme and Triton X-100. The formation of yellow *o*-nitrophenyl (ONP) from *o*-nitrophenyl-β-d-galactopyranoside (ONPG) was measured colorimetrically at 420 nm. The results were expressed as a percentage of WT activity at 25°C.

To assay the phosphatase activity of DesK variants, AKP20 (*desK*::*km-*P*km-desR amyE*::P*des-lacZ*) transformed with pHPKS expressing different DesK variants under the inducible P*xyl* promoter was grown overnight at 37°C in LB. Cells were collected and diluted in the same medium to a final OD_525_ of 0.10 either in the presence or in the absence of 0.05% xylose and grown at 37°C with shaking. β-Galactosidase activities were determined 4 h after dilution. Results were expressed as a percentage of WT activity in the absence of xylose.

### Molecular modeling and simulations.

Protein models were built from X-ray structures of DesKC H188V in PDB entry 3EHJ and H188E in PDB entry 3GIE, as well as from models of TM5-DesK built in our previous work (14, 17). They all encompass residues 126 to 370 of B. subtilis DesK, as in UniProt entry O34757 (i.e., spans from TM5 until the last natural amino acid of the protein; thus, it is termed TM5-DesKC). For atomistic molecular dynamics simulations, the models were embedded in a 1,2-dioleoyl-*sn*-glycero-3-phosphocholine (DOPC) membrane and hydrated with explicit water plus K^+^ and Cl^−^ ions to 0.15 M concentration using the CHARMM-GUI server (37, 38). Inside CHARMM-GUI, the resulting systems also were parameterized using CHARMM27 parameters for the protein, CHARMM36 parameters for lipids, and a TIP3P (39) model for water, totaling around 235,000 atoms per system. The protocol provided by the CHARMM-GUI server was used in the NAMD program to equilibrate (in NVT) the systems up to 303.15 K and 1 atm. Production simulations were then carried out in NPT with NAMD using 2-fs time steps for integration and a cutoff at 12 Å for nonbonded interactions, with a switching function from 10 to 12 Å and particle-mesh Ewald treatment of electrostatics with a grid spacing of 1 Å. Simulations introduced in this work (i.e., on TM5-DesKC_P148A-L174P_ and on TM5-DesKC_P148A_; Fig. 6C and E, respectively) were produced for 200 ns each (in single replicas). Simulations from our previous work (of which representative snapshots are shown in Fig. 6D and F) lasted around 50 ns and are described in detail in reference 17. All snapshots shown in Fig. 6C to F correspond to manually picked snapshots representative of the main events we aim to communicate, while more objective, quantitative data for the two new simulations are given in Fig. S1 in the supplemental material at https://doi.org/10.5281/zenodo.3523363.

## References

[1] Stock AM, Robinson VL, Goudreau PN 2000 Two-component signal transduction. Annu Rev Biochem 69:183–215. doi:10.1146/annurev.biochem.69.1.183. 10966457

[2] Parkinson JS 2010 Signaling mechanisms of HAMP domains in chemoreceptors and sensor kinases. Annu Rev Microbiol 64:101–122. doi:10.1146/annurev.micro.112408.134215. 20690824

[3] Capra EJ, Laub MT 2012 Evolution of two-component signal transduction systems. Annu Rev Microbiol 66:325–347. doi:10.1146/annurev-micro-092611-150039. 22746333 PMC4097194

[4] Matthews EE, Zoonens M, Engelman DM 2006 Dynamic helix interactions in transmembrane signaling. Cell 127:447–450. doi:10.1016/j.cell.2006.10.016. 17081964

[5] Gushchin I, Melnikov I, Polovinkin V, Ishchenko A, Yuzhakova A, Buslaev P, Bourenkov G, Grudinin S, Round E, Balandin T, Borshchevskiy V, Willbold D, Leonard G, Büldt G, Popov A, Gordeliy V 2017 Mechanism of transmembrane signaling by sensor histidine kinases. Science 356:eaah6345. doi:10.1126/science.aah6345. 28522691

[6] Ottemann KM, Xiao W, Shin YK, Koshland DE 1999 A piston model for transmembrane signaling of the aspartate receptor. Science 285:1751–1754. doi:10.1126/science.285.5434.1751. 10481014

[7] Zschiedrich CP, Keidel V, Szurmant H 2016 Molecular mechanisms of two-component signal transduction. J Mol Biol 428:3752–3775. doi:10.1016/j.jmb.2016.08.003. 27519796 PMC5023499

[8] Jacob-Dubuisson F, Mechaly A, Betton J-M, Antoine R 2018 Structural insights into the signalling mechanisms of two-component systems. Nat Rev Microbiol 16:585–593. doi:10.1038/s41579-018-0055-7. 30008469

[9] Gushchin I, Gordeliy V 2018 Transmembrane signal transduction in two-component systems: piston, scissoring, or helical rotation? Bioessays 40:1700197. doi:10.1002/bies.201700197. 29280502

[10] Buschiazzo A, Trajtenberg F 2019 Two-component sensing and regulation: how do histidine kinases talk with response regulators at the molecular level? Annu Rev Microbiol 73:507–528. doi:10.1146/annurev-micro-091018-054627. 31226026

[11] Weber MH, Klein W, Müller L, Niess UM, Marahiel MA 2001 Role of the *Bacillus subtilis* fatty acid desaturase in membrane adaptation during cold shock. Mol Microbiol 39:1321–1329. doi:10.1111/j.1365-2958.2001.02322.x. 11251847

[12] Altabe SG, Aguilar P, Caballero GM, de Mendoza D 2003 The *Bacillus subtilis* acyl lipid desaturase is a Δ5 desaturase. J Bacteriol 185:3228–3231. doi:10.1128/jb.185.10.3228-3231.2003. 12730185 PMC154086

[13] Aguilar PS, Hernandez-Arriaga AM, Cybulski LE, Erazo AC, de Mendoza D 2001 Molecular basis of thermosensing: a two-component signal transduction thermometer in *Bacillus subtilis*. EMBO J 20:1681–1691. doi:10.1093/emboj/20.7.1681. 11285232 PMC145467

[14] Albanesi D, Martín M, Trajtenberg F, Mansilla MC, Haouz A, Alzari PM, de Mendoza D, Buschiazzo A 2009 Structural plasticity and catalysis regulation of a thermosensor histidine kinase. Proc Natl Acad Sci U S A 106:16185–16190. doi:10.1073/pnas.0906699106. 19805278 PMC2738621

[15] Cybulski LE, Martín M, Mansilla MC, Fernández A, de Mendoza D 2010 Membrane thickness cue for cold sensing in a bacterium. Curr Biol 20:1539–1544. doi:10.1016/j.cub.2010.06.074. 20705470

[16] Abriata LA, Albanesi D, Dal Peraro M, de Mendoza D 2017 Signal sensing and transduction by histidine kinases as unveiled through studies on a temperature sensor. Acc Chem Res 50:1359–1366. doi:10.1021/acs.accounts.6b00593. 28475313

[17] Saita E, Abriata LA, Tsai YT, Trajtenberg F, Lemmin T, Buschiazzo A, Dal Peraro M, de Mendoza D, Albanesi D 2015 A coiled coil switch mediates cold sensing by the thermosensory protein DesK. Mol Microbiol 98:258–271. doi:10.1111/mmi.13118. 26172072

[18] Lupas AN, Gruber M 2005 The structure of α-helical coiled coils. Adv Protein Chem 70:37–38. doi:10.1016/S0065-3233(05)70003-6. 15837513

[19] Inda ME, Vazquez DB, Fernández A, Cybulski LE 2019 Reverse engineering of a thermosensing regulator switch. J Mol Biol 431:1016–1024. doi:10.1016/j.jmb.2019.01.025. 30738600

[20] Molnar KS, Bonomi M, Pellarin R, Clinthorne GD, Gonzalez G, Goldberg SD, Goulian M, Sali A, DeGrado WF 2014 Cys-scanning disulfide crosslinking and bayesian modeling probe the transmembrane signaling mechanism of the histidine kinase, PhoQ. Structure 22:1239–1251. doi:10.1016/j.str.2014.04.019. 25087511 PMC4322757

[21] Bhate MP, Molnar KS, Goulian M, DeGrado WF 2015 Signal transduction in histidine kinases: insights from new structures. Structure 23:981–994. doi:10.1016/j.str.2015.04.002. 25982528 PMC4456306

[22] Mechaly AE, Sassoon N, Betton J-M, Alzari PM 2014 Segmental helical motions and dynamical asymmetry modulate histidine kinase autophosphorylation. PLoS Biol 12:e1001776. doi:10.1371/journal.pbio.1001776. 24492262 PMC3904827

[23] Casino P, Miguel-Romero L, Marina A 2014 Visualizing autophosphorylation in histidine kinases. Nat Commun 5:3258. doi:10.1038/ncomms4258. 24500224

[24] Diensthuber RP, Bommer M, Gleichmann T, Möglich A 2013 Full-length structure of a sensor histidine kinase pinpoints coaxial coiled coils as signal transducers and modulators. Structure 21:1127–1136. doi:10.1016/j.str.2013.04.024. 23746806

[25] Lee Y, Nishizawa T, Yamashita K, Ishitani R, Nureki O 2015 Structural basis for the facilitative diffusion mechanism by SemiSWEET transporter. Nat Commun 6:6112. doi:10.1038/ncomms7112. 25598322 PMC4309421

[26] Jacob J, Duclohier H, Cafiso DS 1999 The role of proline and glycine in determining the backbone flexibility of a channel-forming peptide. Biophys J 76:1367–1376. doi:10.1016/S0006-3495(99)77298-X. 10049319 PMC1300115

[27] Chow WY, Forman CJ, Bihan D, Puszkarska AM, Rajan R, Reid DG, Slatter DA, Colwell LJ, Wales DJ, Farndale RW, Duer MJ 2018 Proline provides site-specific flexibility for in vivo collagen. Sci Rep 8:13809. doi:10.1038/s41598-018-31937-x. 30218106 PMC6138679

[28] Kumeta M, Konishi HA, Zhang W, Sakagami S, Yoshimura SH 2018 Prolines in the α-helix confer the structural flexibility and functional integrity of importin-β. J Cell Sci 131:jcs206326. doi:10.1242/jcs.206326. 29142102

[29] Cordes FS, Bright JN, Sansom M 2002 Proline-induced distortions of transmembrane helices. J Mol Biol 323:951–960. doi:10.1016/S0022-2836(02)01006-9. 12417206

[30] Schmidt T, Situ AJ, Ulmer TS 2016 Structural and thermodynamic basis of proline-induced transmembrane complex stabilization. Sci Rep 6:29809. doi:10.1038/srep29809. 27436065 PMC4951694

[31] Kumar S, Das M, Hadad CM, Musier-Forsyth K 2012 Substrate specificity of bacterial prolyl-tRNA synthetase editing domain is controlled by a tunable hydrophobic pocket. J Biol Chem 287:3175–3184. doi:10.1074/jbc.M111.313619. 22128149 PMC3270972

[32] Tieleman DP, Shrivastava IH, Ulmschneider MR, Sansom M 2001 Proline-induced hinges in transmembrane helices: possible roles in ion channel gating. Proteins 44:63–72. doi:10.1002/prot.1073. 11391769

[33] Härtl B, Wehrl W, Wiegert T, Homuth G, Schumann W 2001 Development of a new integration site within the *Bacillus subtilis* chromosome and construction of compatible expression cassettes. J Bacteriol 183:2696–2699. doi:10.1128/JB.183.8.2696-2699.2001. 11274134 PMC95191

[34] Johansson P, Hederstedt L 1999 Organization of genes for tetrapyrrole biosynthesis in Gram-positive bacteria. Microbiology 145:529–538. doi:10.1099/13500872-145-3-529. 10217486

[35] Díaz AR, Porrini L, de Mendoza D, Mansilla MC 2019 A genetic screen for mutations affecting temperature sensing in *Bacillus subtilis*. Microbiology 165:90–101. doi:10.1099/mic.0.000741. 30431418

[36] Beranová J, Mansilla MC, de Mendoza D, Elhottová D, Konopásek I 2010 Differences in cold adaptation of *Bacillus subtilis* under anaerobic and aerobic conditions. J Bacteriol 192:4164–4171. doi:10.1128/JB.00384-10. 20581210 PMC2916416

[37] Jo S, Kim T, Im W 2007 Automated builder and database of protein/membrane complexes for molecular dynamics simulations. PLoS One 2:e880. doi:10.1371/journal.pone.0000880. 17849009 PMC1963319

[38] Jo S, Kim T, Iyer VG, Im W 2008 CHARMM-GUI: a web-based graphical user interface for CHARMM. J Comput Chem 29:1859–1865. doi:10.1002/jcc.20945. 18351591

[39] Jorgensen WL, Chandrasekhar J, Madura JD, Impey RW, Klein ML 1983 Comparison of simple potential functions for simulating liquid water. J Chem Phys 79:926–935. doi:10.1063/1.445869.

